# Conformal Radiotherapy for Squamous Cell Carcinoma of Gallbladder: A Case Report

**DOI:** 10.1155/2010/645172

**Published:** 2010-03-18

**Authors:** Jia-zhou Hou, Zhao-chong Zeng, Jing Sun, Yuan Ji

**Affiliations:** ^1^Department of Radiation Oncology, Zhongshan Hospital, Fudan University, Shanghai 200032, China; ^2^Department of Pathology, Zhongshan Hospital, Fudan University, Shanghai 200032, China

## Abstract

*Background*. Squamous cell carcinoma of the gallbladder is a rare disease with symptoms developing late in its course, so that it often presents as an aggressive tumor with a poor prognosis. *Case report*. We describe a 58-year-old male with a 5-week history of hypodynamia. He was found to have squamous cell carcinoma of the gallbladder with liver invasion and lymph node metastases. He underwent treatment with 3-dimensional conformal radiation therapy (CRT). A follow-up computer tomography (CT) scan showed complete tumor remission 2 months after the completion of CRT. The patient survived for 14 months after the end of treatment and died of multiple liver metastases. *Conclusion*. The efficacy of radiotherapy in this case is encouraging and suggests a potential role for such therapy in similar cases. The benefit in terms of survival warrants further study.

## 1. Background

Primary carcinoma of the gallbladder is the fifth most common malignancy of the digestive tract [1]. Squamous cell carcinoma, however, accounts for less than 3.93% of gallbladder cancers in China [2]. Symptoms generally develop late in the course of the disease, by which time the malignancy is aggressive and carries a poor prognosis. We report a case of advanced stage squamous cell carcinoma of the gallbladder that acquired complete tumor remission 2 months after the completion of conformal radiation therapy (CRT).

## 2. Case Report

**Figure 1:**
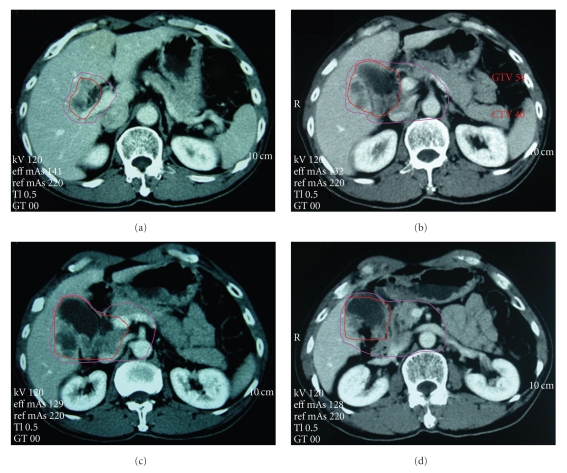
CT sections showing contours before radiotherapy. Radiation ports encompassed (a-b) regions of the liver involved by the gallbladder tumor and (c-d) carcinoma located at the neck of the gallbladder. GTV54 and CTV40 have been shown in the slice. Abbreviation: CTV: clinical target volume with pink lines; GTV: gross tumor volume with red lines.

**Figure 2:**
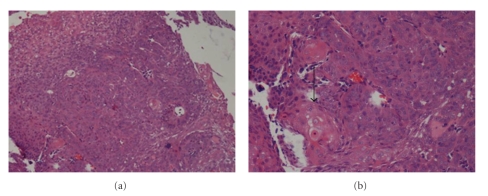
Micrographs of the squamous cell carcinoma of the gallbladder. (a) A liver biopsy showing squamous cell carcinoma with intermediate differentiation under low power magnification (low power field), and (b) a squamatoid pearl (black arrow) under high power magnification.

A 58-year-old male was admitted to the local hospital with hypodynamia for 5 weeks. Physical examination was unremarkable, but ultrasound examination showed liver and gallbladder space-occupying lesions. Then an abdominal computer tomography (CT) scan was recommended and showed a tumor involving the gallbladder and the surrounding liver tissue, with possible portal lymph node metastases and gallstones (Figure 1). For further diagnosis and treatment, the patient was referred to our hospital. Physical examination was still unremarkable, the Karnofsky performance status (KPS) score was 90. A percutaneous biopsy was performed with ultrasound guidance, and the pathological diagnosis was squamous cell carcinoma. The pathologicdifferentiation was grade II (Figure 2). Laboratory tests showed the following: red blood cells 4.5 × 10^9^/L, hemoglobin 153 g/L, white blood cells 5.2 × 10^9^/L, Neutrophils 58.2%, platelets 211 × 10^9^/L, total bilirubin 10.4 mmol/L, alanine aminotransferase 24 U/L, aspartate aminotransferase 31 U/L, alkaline phosphatase 71 U/L, *α*-fetoprotein (AFP) 1.8 *μ*g/L, carbohydrate antigen 19.9 (CA19.9) 8.5 U/L, and carcinoembryonic antigen (CEA) 4.56 U/mL. According to the CT, the surgeons figured that the tumor had lymph node metastases, which involved portal vein lymph node, inferior vena caval lymph node, and peripancreatic lymph node. They considered that the tumor was unresectable. The patient was recommended to receive conformal radiotherapy due to consideration of the advanced nature of the disease.

## 3. Radiotherapy

The patient provided written informed consent regarding treatment. No systemic chemotherapy was added during radiotherapy. He underwent three-dimensional (3D) CRT with limited-field external beam radiation using a linear accelerator with 15 megavoltage photons at an outpatient clinic. For radiotherapy planning, the patient underwent a CT scan in the supine position with both arms raised above the head. The CT images were transferred to a 3D CRT planning system (Pinnacle 7.6C). The gross target volume included the gallbladder tumor, involved liver tissue, and abnormally enlarged portal lymph nodes. The clinical target volume included the gross target volume and the peripancreatic and celiac trunk lymph nodes, which were at risk for metastases. The planning treatment volume included the clinical target volume and 0.7 cm margins for geometric uncertainties. The treatment portals encompassed the planning treatment volume plus 0.7 cm in each direction. Dose-volume histograms of the planning treatment volume, kidneys, liver, and spinal cord were necessary to select the optimal dose distribution plan. Coverage of 99.9% of the planning treatment volume by the 95% isodose line was required. The clinical target volume was subject to 40 Gy, with 14 Gy delivered to the gross tumor volume through reduced fields. The dose was in fractions of 2.0 Gy, once daily, five times per week for 5 weeks.

## 4. Result

During the entire period of radiation therapy, physical examination, routine blood tests, and serum biochemistry tests were performed once a week and remained normal. The patient's hypodynamia was relieved after completion of radiotherapy. The tumor marker levels were similar before and after treatment and considered normal (after treatment: AFP 2.1 ng/mL, CA19.9 7.9 U/mL, CEA 4.1 U/mL).

**Figure 3:**
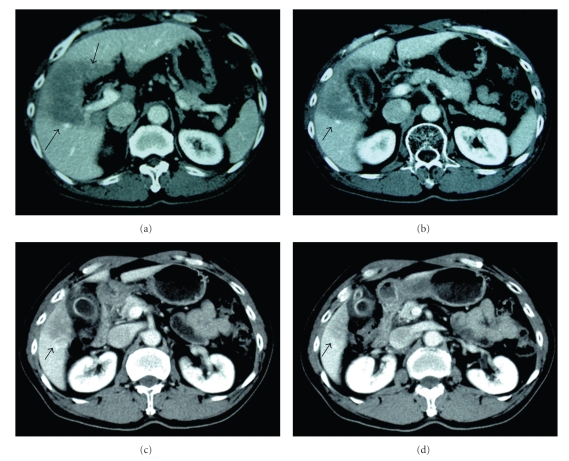
Postradiation CT scans corresponding to the sections in Figure 1. The region of low attenuation in the liver (arrows) corresponded to the treatment volume at 2 months after completion of 3D CRT. The tumor appears to have a complete response to 3D CRT.

The patient was advised to return for follow-up 6 weeks after completion of the radiotherapy. Response to radiotherapy was evaluated at that time with an enhanced abdominal CT (Figure 3). CT showed that the tumor appeared to have a complete response to 3D CRT. A chest radiograph and whole body bone scan showed no evidence of metastasis. The patient was monitored every 3 months thereafter, and the abdominal CT remained unremarkable until 13 months after completion of radiotherapy. At that time, multiple metastatic lesions were found on CT. Two months later, the patient died of liver failure induced by intrahepatic lesions. The survival period was 15 months from initiation of radiation therapy to death.

## 5. Discussion and Conclusion

Gallbladder cancer is the most common malignant originating from biliary tract and represents approximately 8.4% of the estimated cases of hepatobiliary cancers diagnosed in the USA [3]. Squamous cell carcinoma of the gallbladder is a rare disease, accounting for 0.5%–12.7% of all malignant tumors of the gallbladder [4]. This malignancy is an aggressive and late symptomatic disease and most patients are treated at an advanced stage with a poor prognosis.

The only potentially curative therapy for gallbladder carcinoma is surgical resection. Unfortunately, most patients with this type of cancer have unresectable disease—only 10%–30% of patients are candidates for surgery at presentation. At present, no therapy is defined for unresectable cancer of the gallbladder, especially for squamous cell carcinoma. Reports on the efficacy of radiotherapy for gallbladder carcinoma are disappointing; most series have a small number of patients and results regarding survival are inconsistent. However, one study indicated that external beam radiotherapy, as an adjuvant to surgical treatment, has some beneficial effect on survival [5]. Radiotherapy may also be used in palliative management of advanced gallbladder carcinoma. We found no reports on radiotherapy for squamous cell carcinoma of gallbladder. In this case, the patient was treated with radiotherapy alone. CT scans showed complete tumor remission from 2 months to 13 months after treatment. This is encouraging evidence for the efficacy of radiotherapy for squamous cell carcinoma of the gallbladder, suggesting a possible role in treatment. However, the benefit in survival must be studied further. Ultimately, our patient died of liver failure due to intrahepatic metastases. Perhaps the addition of chemotherapy to radiotherapy may have influenced the outcome.

## References

[1] Misra S, Chaturvedi A, C. Misra N, D. Sharma I (2003). Carcinoma of the gallbladder. *Lancet Oncology*.

[2] Zou Q, Zhang L (1999). The national investigation on case of carcinoma of gallbladder. *Chinese Journal of Hepatobiliary Surgery*.

[3] Donohue JH, Stewart AK, Menck HR (1998). The national cancer data base report on carcinoma of the gallbladder, 1989–1995. *Cancer*.

[4] Khaira HS, Awad RW, Thompson AK (1995). Squamous cell carcinoma of the gallbladder presenting with a biliary-colic fistula. *European Journal of Surgical Oncology*.

[5] Houry S, Haccart V, Huguier M, Schlienger M (1999). Gallbladder cancer: role of radiation therapy. *Hepato-Gastroenterology*.

